# Gaseous Hydrogen Sulfide Protects against Myocardial Ischemia-Reperfusion Injury in Mice Partially Independent from Hypometabolism

**DOI:** 10.1371/journal.pone.0063291

**Published:** 2013-05-10

**Authors:** Pauline M. Snijder, Rudolf A. de Boer, Eelke M. Bos, Joost C. van den Born, Willem-Peter T. Ruifrok, Inge Vreeswijk-Baudoin, Marcory C. R. F. van Dijk, Jan-Luuk Hillebrands, Henri G. D. Leuvenink, Harry van Goor

**Affiliations:** 1 Department of Pathology and Medical Biology, University of Groningen, University Medical Center Groningen, Groningen, The Netherlands; 2 Department of Surgery, University of Groningen, University Medical Center Groningen, Groningen, The Netherlands; 3 Department of Cardiology, University of Groningen, University Medical Center Groningen, Groningen, The Netherlands; Virginia Commonwealth University Medical Center, United States of America

## Abstract

**Background:**

Ischemia-reperfusion injury (IRI) is a major cause of cardiac damage following various pathological processes. Gaseous hydrogen sulfide (H_2_S) is protective during IRI by inducing a hypometabolic state in mice which is associated with anti-apoptotic, anti-inflammatory and antioxidant properties. We investigated whether gaseous H_2_S administration is protective in cardiac IRI and whether non-hypometabolic concentrations of H_2_S have similar protective properties.

**Methods:**

Male C57BL/6 mice received a 0, 10, or 100 ppm H_2_S-N_2_ mixture starting 30 minutes prior to ischemia until 5 minutes pre-reperfusion. IRI was inflicted by temporary ligation of the left coronary artery for 30 minutes. High-resolution respirometry equipment was used to assess CO_2_-production and blood pressure was measured using internal transmitters. The effects of H_2_S were assessed by histological and molecular analysis.

**Results:**

Treatment with 100 ppm H_2_S decreased CO_2_-production by 72%, blood pressure by 14% and heart rate by 25%, while treatment with 10 ppm H_2_S had no effects. At day 1 of reperfusion 10 ppm H_2_S showed no effect on necrosis, while treatment with 100 ppm H_2_S reduced necrosis by 62% (p<0.05). Seven days post-reperfusion, both 10 ppm (p<0.01) and 100 ppm (p<0.05) H_2_S showed a reduction in fibrosis compared to IRI animals. Both 10 ppm and 100 ppm H_2_S reduced granulocyte-influx by 43% (p<0.05) and 60% (p<0.001), respectively. At 7 days post-reperfusion both 10 and 100 ppm H_2_S reduced expression of fibronectin by 63% (p<0.05) and 67% (p<0.01) and ANP by 84% and 63% (p<0.05), respectively.

**Conclusions:**

Gaseous administration of H_2_S is protective when administered during a cardiac ischemic insult. Although hypometabolism is restricted to small animals, we now showed that low non-hypometabolic concentrations of H_2_S also have protective properties in IRI. Since IRI is a frequent cause of myocardial damage during percutaneous coronary intervention and cardiac transplantation, H_2_S treatment might lead to novel therapeutical modalities.

## Introduction

Ischemia-reperfusion injury (IRI) is the most important cause of myocardial damage and subsequent heart failure. Although IRI is most frequently caused by acute myocardial infarction with (early or late) reperfusion, it can also be observed following surgical procedures such as cardiopulmonary bypass or cardiac transplantation. [1], [2] Myocardial IRI causes acute tissue responses characterized by inflammation and upregulation of inflammatory mediators. This process ultimately leads to irreversible fibrotic damage. [3], [4] Despite major therapeutical developments, cardiovascular disease remains the leading cause of death in the western world [5].

Hydrogen sulfide (H_2_S) has drawn considerable attention for its role in various (patho)physiological processes. It is, in addition to nitric oxide and carbon monoxide, acknowledged as the third gasotransmitter, sharing many functions with these gases. [6] H_2_S is endogenously produced and exerts fine, modulatory control over cellular functions by influencing an array of intracellular signaling processes. H_2_S-producing enzymes and H_2_S-plasma levels are reduced in various diseases.[7]–[9] Exogenously administered H_2_S can reversibly induce a hypometabolic state in mice, during which it rapidly reduces O_2_-consumption, CO_2_-production, core body temperature, heart rate and breathing frequency. [10], [11] The most probable mechanism for these properties is the reversible inhibition of mitochondrial O_2_-consumption and ATP-production through non-permanent binding of sulfide to the terminal enzyme in the electron transport chain, cytochrome c oxidase (complex IV). [12] It was thought that the reduced demand for oxygen during hypometabolism might be one of the protective mechanisms during ischemia. However, H_2_S is also considered protective during other processes critically involved in myocardial IRI such as oxidation, inflammation and apoptosis. These cytoprotective features of H_2_S make it an attractive candidate for therapeutic reduction of the damaging effects of hypoxia [13], [14].

The influence of gaseous administration of H_2_S and the effects of hypometabolic and non-hypometabolic concentrations on the outcome of myocardial IRI remains to be elucidated. Some studies have explored the beneficial effects of soluble H_2_S donors such as NaHS and Na_2_S in myocardial IRI and other models of cardiac damage.[15]–[18] The preference for gaseous administration above injection with H_2_S donors lies within accurate management of the concentration. As opposed to injection with soluble H_2_S donors, gaseous H_2_S is less difficult to dose and has a short wash-out period, leaving its positive effects behind. [11] Moreover, gaseous administration has proven to induce a hypometabolic state, while this has not been shown for intra-peritoneal or intra-venous administration of soluble H_2_S. [10], [11] Although H_2_S does not appear to have hypometabolic effects in ambiently cooled large mammals, thereby questioning its therapeutic applications in humans, the benefical effects of non-hypometabolic concentrations of H_2_S have not been studied. [19], [20] Since minimizing myocardial IRI has broad clinical implications and may have beneficial effects on cardiac surgical outcomes [1], we investigated whether gaseous H_2_S-treatment attenuates myocardial IRI in mice and whether non-hypometabolic concentrations exhibit similar protective properties.

## Materials and Methods

### Ethics Statement

Procedures were in agreement with institutional and legislator regulations and approved by the Committee on the Ethics of Animal Experiments of the University Medical Center Groningen (DEC 5276).Utmost effort was utilized to prevent suffering and minimize the numbers of mice required for each experiment.

### Animals

Male C57BL/6 mice (6–8 weeks, Harlan, Zeist, the Netherlands) were housed at our animal research facility under standard conditions with a 12 h light:dark cycle with free access to water and chow.

### Telemetry

Blood pressure was measured telemetrically (n = 4) using transmitters (TA11PA-C10; Data Sciences International, St Paul, MN, USA). Devices were placed through a midline abdominal incision under anesthesia (2% Isoflurane) and mice were placed on a heating pad to maintain body temperature at 37°C. The catheter was placed in the aorta and the transmitter body in the abdominal cavity. Animals recovered 7 days before commencing measurements. Data were recorded as 10-second averages every minute using Dataquest ART data acquisition system (Data Sciences International). Animals were treated with room air or a H_2_S/air mixture in our respirometry system during measurements. For comparison of blood pressure and heart rate, the average of 20 minutes baseline measurement and 20 minutes of 10 ppm and 100 ppm H_2_S treatment was determined. A crossover design was used in which all animals received all treatments in randomized order.

### Respirometry

Measurement of CO_2_-production was performed as described. [21] Compressed air and 500 ppm H_2_S/N_2_ (Air Products, Amsterdam, the Netherlands) were mixed in a 4∶1 ratio and in a 49∶1 ratio resulting in a 100 ppm H_2_S/17% O_2_ mixture and a 10 ppm H_2_S/17% O_2_ mixture, respectively. CO_2_-production was corrected for body weight and normalized to mean control values. Animals (n = 11) were treated in a crossover model in randomized order and all received room air, 10 ppm H_2_S and 100 ppm H_2_S on different days. Baseline CO_2_ measurements with room air were performed for 30 minutes followed by treatment with either a 10 or 100 ppm H_2_S/17% O_2_ mixture for 30 minutes. Recovery with room air was measured for 30 minutes.

### Myocardial Ischemia-reperfusion and H_2_S Treatment

Mice were intubated and mechanically ventilated (n = 77) with an O_2_/N_2_ mixture in a 4∶1 ratio, an O_2_/100 ppm H_2_S/N_2_ mixture in a 4∶1 ratio or a O_2_/10 ppm H_2_S/N_2_ mixture in a 49∶1 ratio at a frequency of 180/min with a tidal volume of 250 µl using a rodent ventilator (Harvard Midivent model 849). Treatment regimens (Sham n = 15; IRI n = 20; 10 ppm n = 21; 100 ppm n = 21) were randomly assigned and started 30 minutes prior to ischemia until 5 minutes pre-reperfusion. Myocardial IRI was inflicted by temporary ligation of the left anterior descending coronary artery (6.0 prolene suture) for 30 minutes through an incision in the fourth intercostal space under anesthesia (75 mg/kg ketamine, 1 mg/kg domitor). After removing the ligature the heart was inspected for restoration of blood flow and muscle and skin layers were sutured with 5.0 vicryl. Body temperature was monitored with a rectal probe and maintained at 37°C using heat pads. Sham operated animals underwent the same procedure, except the placement of the ligature. Post-operatively, all mice received a subcutaneous injection of 50 µg/kg buprenorphin (Schering-Plough) for analgesic purposes and were allowed to recover from surgery at 37°C in a ventilated incubator. After 1 and 7 days mice were anaesthetized with 2% isoflurane in O_2_ for collection of blood and organs. Blood was collected in heparin containing tubes, centrifuged for 10 minutes at 1000 rcf and plasma was collected and stored at −80°C. The hearts were rapidly excised and mid-papillary slices were fixed in 4% paraformaldehyde, paraffin-embedded and sections were cut for immunohistochemical analysis. Apical parts of the heart were snap frozen in liquid nitrogen and stored at −80°C for molecular analysis.

### Serum Analysis

Cardiac damage was assessed by measuring high sensitive (hs) Troponin-T in serum samples using a standard electrochemiluminescence immunoassay (Roche) in the clinical chemical laboratory.

### Histopathological Scoring

At 1 day of reperfusion the extent of necrosis was determined in haematoxylin-eosin stained sections. At 7 days of reperfusion the extent of fibrosis was determined in Masson stained sections. Both were examined in a blinded fashion. Sections were scanned using an Aperio ScanScope GS (Aperio Technologies, Vista, CA, USA). Total cardiac area, necrotic cardiac area and fibrotic cardiac area were determined using Aperio Imagescope software, and the ratio of necrotic cardiac surface area and fibrotic surface area to total cardiac surface area were determined. Representative photomicrographs were artificially colored indicating the extent of damage [11], [21].

### Immunohistochemistry for Ly-6G

For granulocytes, paraffin-embedded sections were stained for Ly-6G using rat-anti-mouse Ly6G/C-FITC IgG2b antibody (AbCam, Cambridge, MA, USA), followed by rabbit-anti-FITC and HRP-conjugated goat-anti-rabbit antibodies. Slides were scanned using an Aperio ScanScope GL (Aperio Technologies, Vista, CA, USA) and analyzed for positive pixel area (Ly-6G) using the Aperio Positive Pixel Analysis v9.1 algorithm.

### Qualitative Real-Time Polymerase Chain Reaction

RNA extraction, DNAase treatment [21] and cDNA synthesis [22] were performed as described. A relative quantification PCR was performed to determine gene expression (Applied Biosystems, Foster City, CA). β-actin and GAPDH were used as housekeeping genes. The primers used were: fibronectin (NM_010233.1) – Forward: AGGAAATGTACTGAATGCTAGTACCCA, Reverse TCAGATGGCAAAAGAAAGCAGA; ANP (NM_008725.2) – Forward: ACCCTCCTGGAGCTGCG, Reverse: ACCCCACTAGACCACTCATCTACAT; NOX2 (NM_007807.4) – Forward: GATGCAATAAGACTAGGCACAAACC, Reverse: CCATCTCATAACCAGAATAACTCAGGATA; NOX4 (NM_015760.4) – Forward: TGCACCAAACACAGAAGCACA, Reverse: AGCAGGGTATCACTCCATGAATTC. PCR was performed in a volume of 20 µl containing 10 ng cDNA and 15 µl PCR mastermix (SYBR GREEN Applied Biosystems; 5 ml P/N 4309155). The Thermal Profile was performed as described. [22] The average Ct-values for fibronectin, ANP, NOX2 and NOX4 were subtracted from the average β-actin Ct-values and the average of β-actin and GAPDH Ct-values to yield the delta Ct. Results were expressed as 2^−ΔCt^.

### Cell Culture

The H9c2 cell line (ATCC) is an immortalized line with characteristics of rat heart myoblasts. Cells were cultured in Dulbecco’s modified Eagle’s medium (DMEM; Lonza, Germany) containing 4.5 g/l glucose, 10% fetal calf serum (FCS; Bodinco, Alkmaar, the Netherlands), L-glutamine and penicillin (100 U/ml) streptomycin (100 µg/ml) (Lonza, Germany). Cells were cultured using 75 cm^2^ collagen coated flasks (Corning, Schiphol-Rijk, Netherlands) in a humidified atmosphere of 5% CO_2_ and 95% O_2_ at 37°C.

### *In vitro* Model of Oxidative Stress

H9c2 cells grown to 80–90% confluency were harvested using 3 ml trypsin EDTA (200 mg/l) after washing twice with Hank’s Buffered Salt Solution (HBSS) (Lonza; Germany). Cells were cultured in a 24-well plate at a density of ∼10.000 cells/well in 0.5 ml medium. After 24 hours cells were loaded with 15 uM Dihydroethidine (DHE). Culture plates were placed in a humidified chamber with 5% CO_2_ on an automated inverted fluorescent microscope system (TissueFAXS system, TissueGnostics GMBH, Vienna, Austria) which makes sequential photomicrographs of 9 area’s in each well every 5 minutes. After baseline measurements, cells were exposed to Antimycin (50 ug/mL) and NaHS (donor of H_2_S in solution) in a concentration of 1 mM. Fluorescence intensity of every cell was analyzed using the TissueQuest software (TissueGnostics).

### Statistical Analysis

Data were analyzed using GraphPad PRISM 5.0 (GraphPad, San Diego, CA, USA) using two-way ANOVA, Mann-Whitney U, Friedman or Kruskall Wallis tests where appropriate. Bonferroni or Dunns post-hoc analysis were applied where multiple comparisons were made. Normality was tested using the Kolmogorov–Smirnov test. p<0.05 was considered statistically significant. All data are expressed as mean ± SEM (Standard Error of the Mean) unless otherwise indicated.

## Results

### Effect of H_2_S on CO_2_ Production, Blood Pressure and Heart Rate

**Figure 1:**
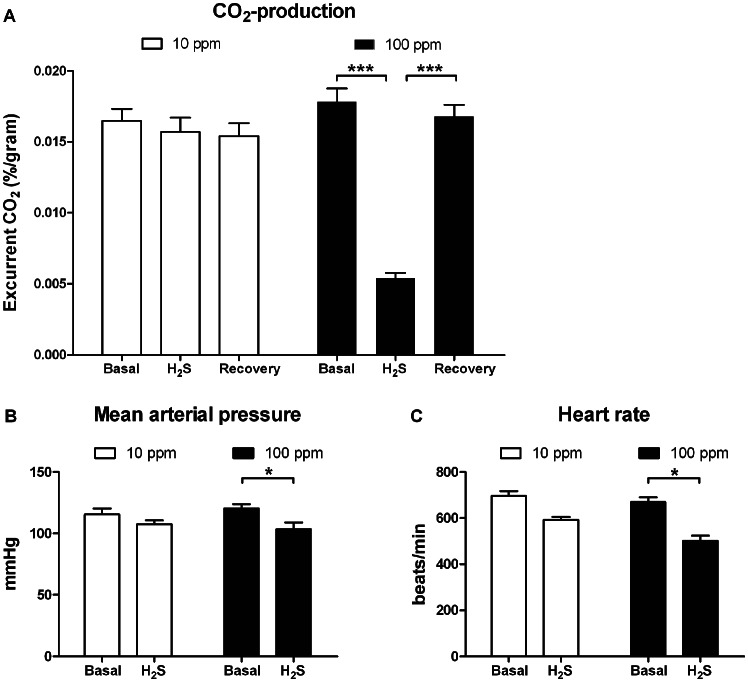
High concentrations of H_2_S induce a state of hypometabolism. Within 15 minutes (A) CO_2_-production decreased by 72% (***p<0.001) in mice (n = 11) subjected to 100 ppm H_2_S. Exposure to 10 ppm H_2_S (n = 11) did not induce a reduction in CO_2_-production in these animals. Cessation of gaseous H_2_S resulted in rapid recovery, within 30 minutes CO_2_-levels returned back to baseline concentrations (***p<0.001). Administration of 100 ppm H_2_S (n = 4) resulted in a 14% decrease in (B) mean arterial pressure and a 25% decrease in (C) heart rate (*p<0.05). However exposure to 10 ppm H_2_S (n = 4) had no effect on mean arterial pressure or heart rate.

Within 15 minutes of treatment with 100 ppm H_2_S induced a state of hypometabolism, concomitant with a reduction in CO_2_-production by an average of 72% compared to basal levels (p<0.001). Cessation of H_2_S resulted in a rapid recovery of CO_2_-production, where CO_2_ concentrations raised to basal levels within 30 minutes (p<0.001). 100 ppm H_2_S lowered blood pressure with 14% (103 *vs.* 120 mmHg, p<0.05) and heart rate with 25% compared to baseline (502 *vs.* 670 beats per minute, p<0.05) 10 ppm H_2_S had no effect on CO_2_ production, blood pressure and heart rate (Figure 1).

### H_2_S Reduces Myocardial Damage

**Figure 2:**
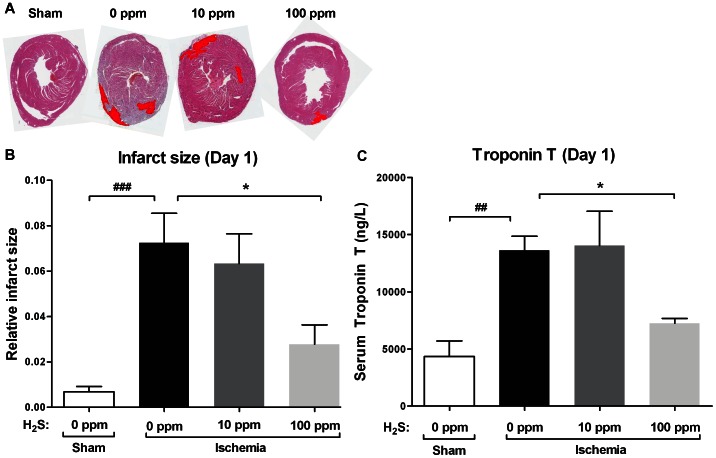
Cardiac damage is reduced by 100 ppm H _2_ **S at 1 day of reperfusion.** (A) Representative photomicrographs of haematoxylin-eosin stained cardiac sections with necrotic area artificially colored red, indicating the extent of necrotic damage found in each group at 1 day of reperfusion. (B) Cardiac IR induced a significant amount of necrosis in IRI animals exposed to 0 ppm H_2_S (^###^p<0.001 vs. sham). In animals treated with 100 ppm H_2_S necrosis was reduced by 62% (*p<0.05 vs. IRI) where as 10 ppm H_2_S had no effect on necrosis. (C) At 1 day of reperfusion hs Troponin-T levels were elevated in IRI animals exposed to 0 ppm H_2_S (^##^p<0.01 vs. sham). In the 100 ppm H_2_S treated group hs Troponin-T levels were reduced compared to 0 ppm treated animals (*p<0.05), 10 ppm H_2_S had no effect.

**Figure 3:**
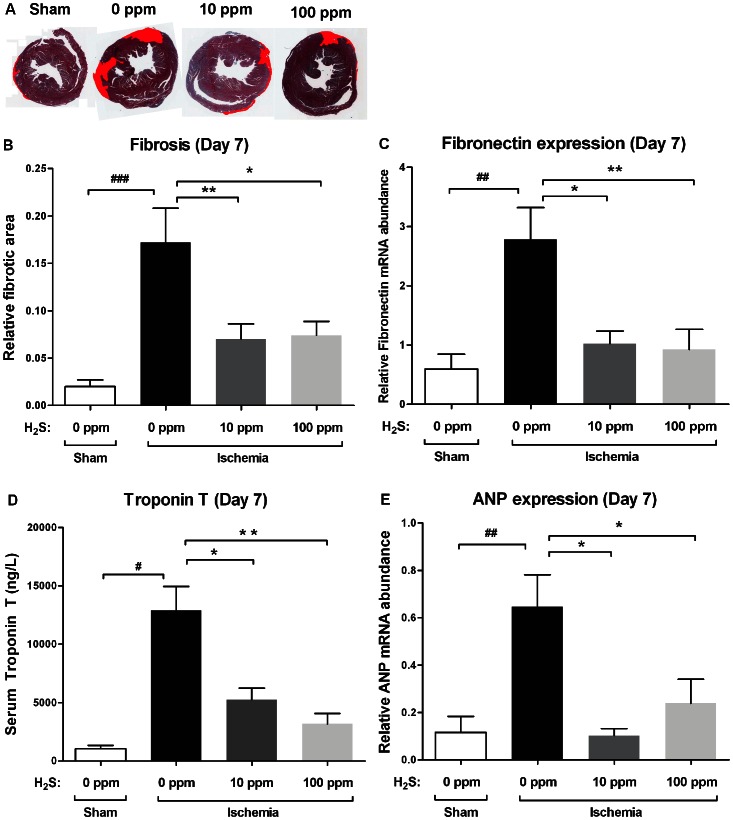
Cardiac damage is reduced by 10 and 100 ppm H_2_S at 7 days of reperfusion. (A) Representative photomicrographs of Masson stained cardiac sections with fibrotic area artificially colored red, indicating the extent of fibrotic damage found in each group at 7 days of reperfusion. (B) Cardiac IR induced a significant amount of fibrosis in IRI animals exposed to 0 ppm H_2_S (^###^p<0.001 vs. sham). In animals treated with 10 ppm and 100 ppm H_2_S fibrosis was significantly reduced (**p<0.01, *p<0.05 vs. IRI). (C) Expression of fibronectin at 7 days of reperfusion was increased in IRI animals (^##^p<0.01 vs. sham). Treatment with 10 and 100 ppm H_2_S reduced the expression of fibronectin (*p<0.05, **p<0.01 vs. IRI). (D) Seven days post-reperfusion hs Troponin-T levels were elevated in IRI animals exposed to 0 ppm H_2_S (^#^p<0.01 vs. sham). Both 10 and 100 ppm H_2_S reduced hs Troponin-T levels by 59% and 75%, respectively, as compared to animals treated with 0 ppm (*p<0.05, **p<0.01 vs. IRI). (E) Expression of ANP mRNA at 7 days of reperfusion was increased in IRI animals (^##^p<0.01 vs. sham). Treatment with 10 and 100 ppm H_2_S reduced the expression of ANP (*p<0.05 vs. IRI).

At 1 day of reperfusion cardiac IRI induced significant necrosis (Figure 2A) in animals exposed to 0 ppm H_2_S when compared to sham animals (p<0.001) as indicated by infarct size. 10 ppm H_2_S did not reduce the size of the necrotic area, while 100 ppm H_2_S reduced infarct size by 62% (p<0.05) (Figure 2B). In mice treated with 10 ppm H_2_S hs Troponin-T levels were not reduced 1 day post-reperfusion, while 100 ppm H_2_S reduced hs Troponin-T levels by 47% (p<0.05) compared to IRI animals (Figure 2C). Fibrosis, as measured by collagen deposition in Masson stained sections at 7 days of reperfusion, was markedly increased in animals treated with 0 ppm H_2_S when compared to sham-operated animals (p<0.001). Treatment with either 10 or 100 ppm of H_2_S reduced collagen deposition to comparable levels (10 ppm: 59%, p<0.01; 100 ppm: 57%, p<0.05) (Figure 3A,B). Cardiac mRNA levels of fibronectin, a marker of myocardial fibrosis, were massively increased in 0 ppm H_2_S treated animals (p<0.01), while no increase was detected in animals of both H_2_S treated groups (10 ppm H_2_S p<0.05; 100 ppm H_2_S p<0.01) (Figure 3C). Seven days post-reperfusion hs Troponin-T levels were reduced by 59% (p<0.05) and 75% (p<0.01) in 10 ppm and 100 ppm H_2_S treated mice, respectively (Figure 3D).

### ANP-gene Expression

mRNA expression of atrial natriuretic peptide (ANP), a marker for induction of the fetal gene program, was significantly increased in hearts of mice treated with 0 ppm H_2_S compared to sham-operated animals at 7 days of reperfusion. In hearts of 10 and 100 ppm H_2_S treated mice the relative ANP expression was significantly reduced compared to mice treated with 0 ppm H_2_S (p<0.05) (Figure 3E).

### Inflammation

**Figure 4:**
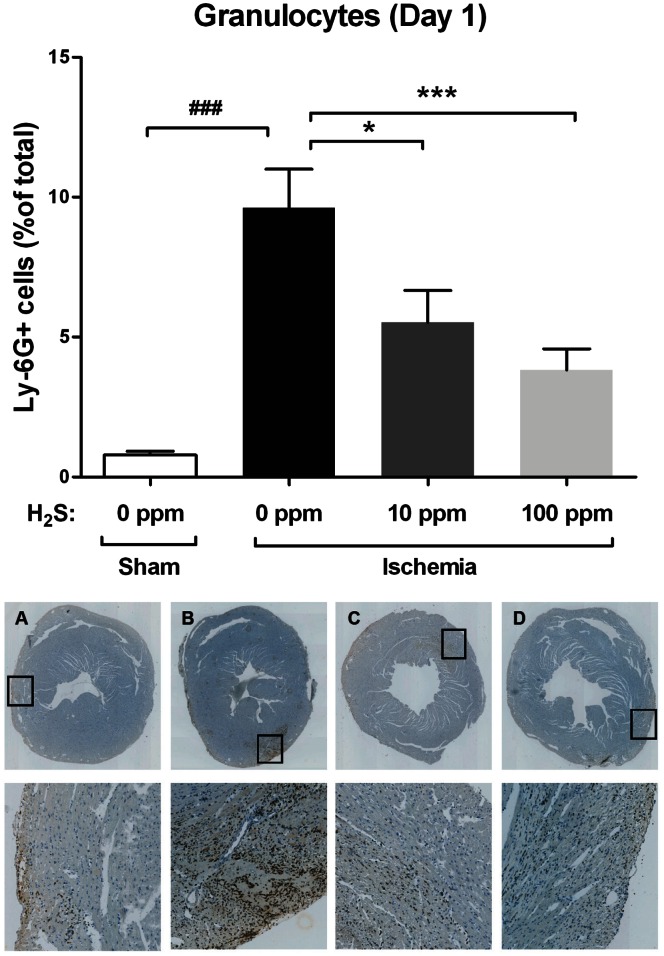
H_2_S reduces cardiac IRI induced inflammation. There was a marked increase in granulocyte influx after cardiac ischemia (p<0.001) compared to sham animals. Exposure to 10 ppm and 100 ppm H_2_S significantly reduced the influx of granulocytes in the infarct area (10 ppm p<0.05; 100 pm p<0.001). Below are representative images of these stainings: (A) Sham (B) IRI, 0 ppm H_2_S (C) IRI, 10 ppm H_2_S (D) IRI, 100 ppm H_2_S.

One day post-reperfusion, Ly-6G-positive granulocytes were increased 12-fold in animals treated with 0 ppm H_2_S compared to sham-operated animals (p<0.001). Exposure to 10 ppm and 100 ppm H_2_S reduced granulocytes by 43% (p<0.05) and 60% (p<0.001), respectively (Figure 4).

### NOX2 and NOX4- Gene Expression

**Figure 5:**
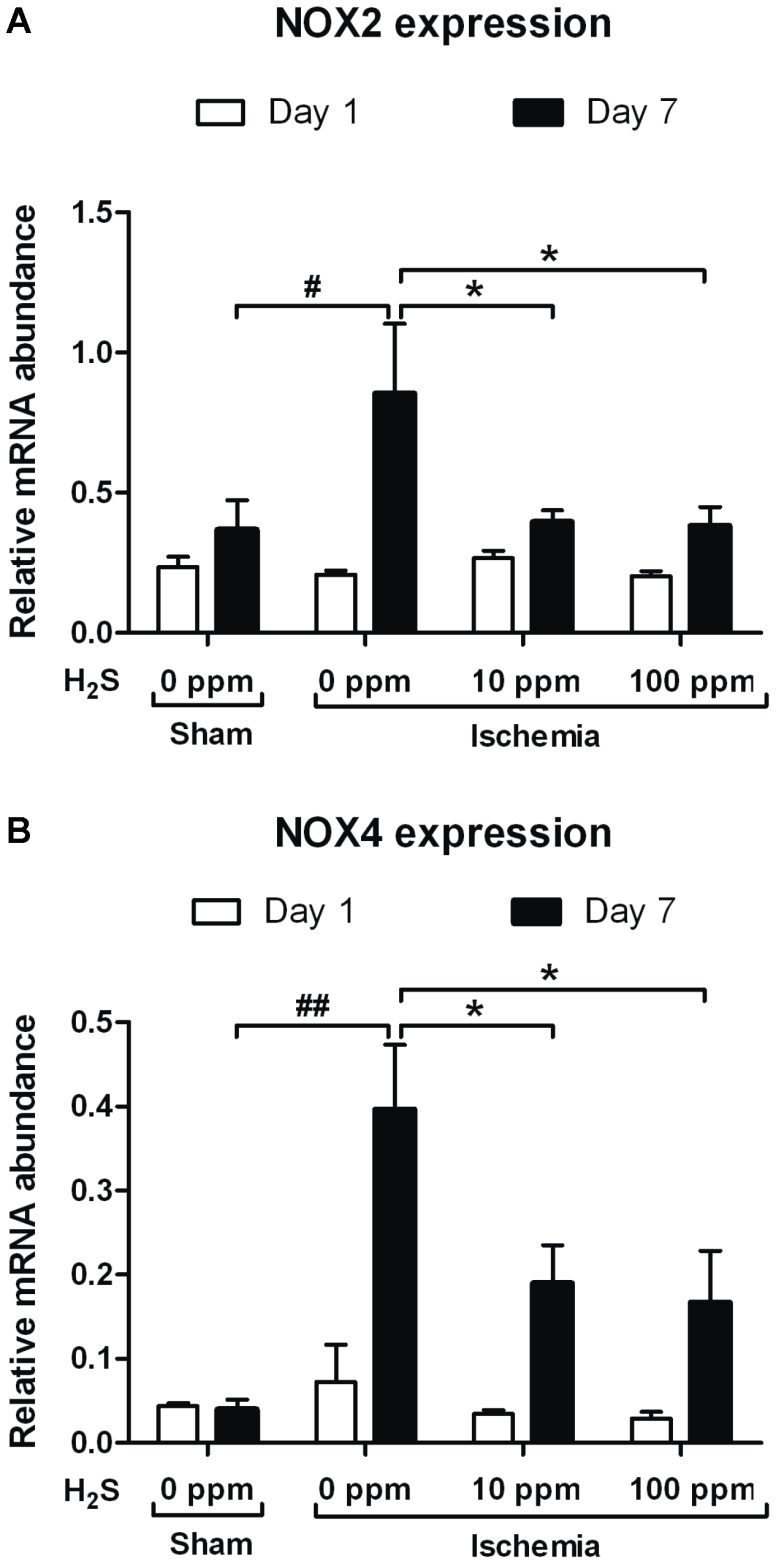
H_2_S attenuates NOX2 and NOX4 upregulation. Expression of (A) NOX2 and (B) NOX4 mRNA was increased in IRI animals at 7 days post-reperfusion (NOX2: ^#^p<0.05, NOX4: ^##^p<0.01 vs. sham). Treatment with 10 and 100 ppm H_2_S reduced the expression of both genes (*p<0.05 vs. IRI). After 1 day of reperfusion no differences were observed between all groups.

To investigate ROS-related genes in vivo, we measured mRNA expression of nicotinamide adenine dinucleotide phosphate oxidase 2 and 4 (NOX2 and NOX4). At 1 day of reperfusion no significant differences were found in NOX2 and NOX4 mRNA expression. Seven days post-reperfusion, NOX2 and NOX4 expression were significantly increased in hearts of mice treated with 0 ppm H_2_S compared to sham operated animals (NOX2: p<0.05, NOX4: p<0.01). NOX2 and NOX4 expression were not amplified in hearts of mice treated with 10 and 100 ppm H_2_S compared to mice treated with 0 ppm H_2_S (p<0.05) (Figure 5A and B).

### Effect of H_2_S on ROS Production *in vitro*

**Figure 6:**
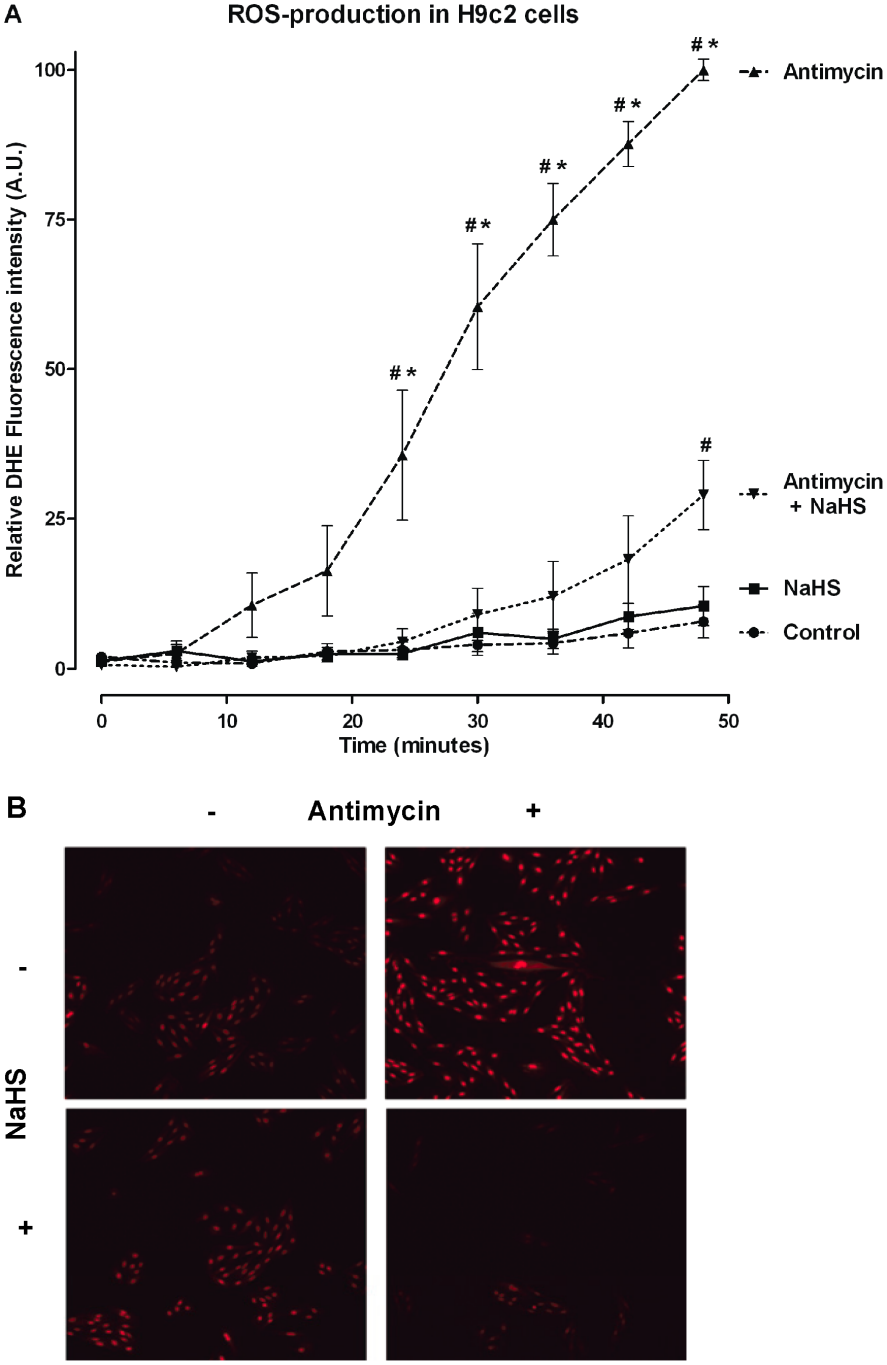
H_2_S reduces ROS production in cultured cardiomyoblasts. (A) Antimycin (50 µg/ml) significantly induced ROS production in H9c2 cells from 24 minutes onwards compared to untreated control cells. Addition of 1 mM NaHS prevented the increase in ROS production. (^#^p<0.001 vs. control; *p<0.001 vs. NaHS) (B) Representative photomicrographs of DHE stained H9c2 cells treated with antimycin and NaHS showing less DHE staining (red) in the NaHS treated cells as compared to cells treated with only antimycin.

Antimycin A induced ROS production in cultured H9c2 rat cardiomyoblasts was significantly reduced by treatment with NaHS. Live cell imaging of DHE fluorescence showed a massive increase in cytoplasmatic ROS production during treatment with Antimycin, whereas addition of NaHS to the medium markedly reduced this fluorescence signal (p<0.001) (Figure 6).

## Discussion

The major finding of this study is that administration of hypometabolic concentrations of gaseous H_2_S during myocardial IR limits the extent of myocardial damage. Furthermore, non-hypometabolic concentrations of H_2_S do not seem protective in the early phase after myocardial infarction, but attenuate ischemia associated processes such as fibrosis and ROS formation. Gaseous administration of H_2_S appears to be an effective way to attenuate the outcome of myocardial IRI, with multiple mechanisms seemingly underlying the protective properties.

H_2_S is cytoprotective during hypoxia in multiple organs. Beneficial effects of H_2_S treatment have been reported in models of shock [23] and intestinal- [24], pulmonary [25], hepatic- [21], [26] and renal ischemia. [11] The cardio-protective effects of H_2_S have been demonstrated in models of myocardial injury. However, most of these studies use injection with soluble H_2_S donor compounds such as sodium hydrosulfide (NaHS) or sodium sulfide (Na_2_S), while no results have been published on gaseous H_2_S.[15]–[18], [27] Gaseous administration might be applicable in patients who are being ventilated because of cardiogenic shock, a state of brain dead or during transplantation procedures. Inhaled H_2_S has beneficial effects in endotoxin induced systemic inflammation [28] and in experimental Parkinson’s disease. [29] We previously showed protective effects of gaseous H_2_S during renal and hepatic IRI. [11], [21] The benefits of gaseous administration compared to injections with soluble H_2_S donors lay within the management of the concentration. As opposed to injection with H_2_S donors it is possible to administer the gas continuously with a stable dose over longer periods of time. Furthermore, when treatment is stopped the effects vanish rapidly while leaving its positive therapeutic effects behind. Also, gaseous administration at higher concentrations has proven to induce a hypometabolic, hibernation-like state in small animals like rodents [10], [11].

As previously shown, 100 ppm H_2_S induces a hypometabolic state and lowers blood pressure, heart rate and CO_2_-production, whereas 10 ppm H_2_S does not. [10], [11], [30] A suspended animation-like state induced by H_2_S protects mice from lethal hypoxia for periods up to 6 hours [31], suggesting that the induction of regulated, reversible and well-controlled hypometabolism in organs holds clinical promise in ischemia-reperfusion related damage where oxygen demand exceeds oxygen availability. Although there was no difference in cardiac necrosis between the 10 ppm treated group and 0 ppm treated group, treatment with 100 ppm significantly reduced necrosis at 1 day of reperfusion. hs Troponin-T levels in serum were in line with this finding. This suggests that H_2_S in non-hypometabolic concentrations is not effective in preventing short-term necrosis caused by ischemia, and that the additional value of H_2_S-induced hypometabolism lies in the early phase of IRI. Although, the protective effects of 100 ppm H_2_S on necrosis might also be caused by a larger amount of H_2_S leading to increased anti-oxidant effects, it is difficult to distinguish between the effects of hypometabolism and other effects attributed to a higher dose. However, inducing a suspended animation-like state might be restricted to small animals like rodents. The applicability of hypometabolism in larger animals is still under debate and we are far from developing therapeutic applications in reducing metabolic rate in the clinical setting with the use of H_2_S [19], [20], [32], [33].

There are a number of potential mechanisms through which H_2_S may exert its cardioprotective effects. Both 10 and 100 ppm of H_2_S were proven anti-inflammatory as evidenced by reduced granulocyte influx into necrotic areas. Treatment with H_2_S also lowered the influx of granulocytes after renal IRI. [11] Furthermore, H_2_S inhibits neutrophil adhesion and activation in response to inflammatory stimuli and suppresses the release of the pro-inflammatory mediator tumor necrosis factor-alpha. [34], [35] Other studies report that H_2_S mediates pro-inflammatory effects by potentiating sulfide production in neutrophils [36] and mediating leukocyte activation. [37] Although granulocyte influx seems to be reduced by treatment with H_2_S, literature is inconclusive on the contribution of neutrophil invasion to final myocardial infarct size and appears not to be a dominant factor [38].

We show that treatment with H_2_S protects against fibrosis at day 7 of reperfusion, as evidenced by reduced collagen deposition and fibronectin expression. Interestingly, the amount of necrosis differs between 10 and 100 ppm H_2_S at day 1 of reperfusion, but this does not translate into differences in fibrotic area size after 7 days. This indicates that treatment with both concentrations of H_2_S attenuate the onset of fibrosis. The prevention of fibrosis is in accordance with previous literature showing decreased cardiac remodelling and fibrosis in models of myocardial infarction and heart failure after H_2_S treatment. [15], [39], [40] Although we find reduced fibrosis with both concentrations of H_2_S, a balanced development of fibrosis remains essential. Suppressed fibrosis with no reduction in the extent of necrosis predisposes to infarct expansion and tissue rupture. [41] Since 10 ppm H_2_S does not affect necrosis 1 day post-reperfusion, the anti-fibrotic effects at day 7 of reperfusion are not beneficial per se.

Another functional property of H_2_S relates to the inhibition of ROS production, since the imbalance in redox status and oxidative stress contributes to fibrosis. [42] ROS-generating NOX2 and NOX4 are both increased after ischemic events in experimental models and their deficiency is protective in these models. [43], [44] Seven days post-reperfusion, we found attenuated expression of NOX2 and NOX4 in both H_2_S treated groups indicating less ROS production in vivo. We did not find an alteration of these genes at 1 day of reperfusion, which is in concordance with previous literature concerning NOX2. [45] Although it is not possible to distinguish whether these components originate from the myocardium or from phagocytes migrated into the myocardium, these results point towards increased oxidative stress in the infarcted heart, and a possible beneficial involvement for the effects of H_2_S at the later time point. Furthermore, ROS production was markedly reduced in H_2_S treated cardiomyoblasts in an *in vitro* model of Antimycin induced oxidative stress, indicating direct scavenging or reduction in production of ROS by mitochondria. H_2_S has direct scavenging effects on ROS, but also has indirect effects via activation of antioxidant mechanisms, such as increasing glutathione levels. [46], [47] Another mechanism that could be involved is the capacity of H_2_S to modulate cellular respiration, as the inhibition of mitochondrial respiration has been shown to protect against myocardial IRI by limiting ROS production in mitochondria. [48] Antioxidant effects of H_2_S may be of critical importance for the treatment of myocardial IRI because oxidative stress plays a prominent role in the development of cardiac damage and remodeling [42].

The effect of exogenous H_2_S on blood pressure is still under debate. *In vivo* and *ex vivo* studies revealed conflicting responses to H_2_S treatment.[49]–[53] The effects of H_2_S on heart rate are also ambiguous; ranging from no change [54] to decreased heart rate in others. [50] Ufnal et al. noticed an increased heart rate upon NaHS infusion, however dependent on H_2_S concentration in cerebrospinal fluid. [55] In additional support of this last view, suppression of H_2_S production either pharmacologically [56] or genetically [57] leads to an increase in blood pressure. These opposing results might be attributable to differences in dose and route of administration.

In this study we show that 100 ppm of gaseous H_2_S significantly lowers blood pressure and heart rate which might have affected cardiac workload and oxygen demand. Since we did not add a group with similar decrease in heart rate and blood pressure or a hypometabolic group with normal heart rate and blood pressure, we can not exclude this phenomenon to be responsible for the improved outcome. Aside from other protective effects of H_2_S, it is thought that the reduced demand for oxygen during hypometabolism might be one of the protective mechanisms during ischemia based on the fact that oxygen availability and oxygen expenditure are more balanced. On the other hand the protective effects can not solely be explained by these effects since 10 ppm H_2_S does not alter heart rate and blood pressure and has positive effects on several damage parameters. Another approach might be local delivery of H_2_S by H_2_S-donors thereby circumventing its systemic effects, which has previously been shown to be protective. [15] However, the highly volatile nature of H_2_S and the associated difficulties in measuring this compound make it difficult to determine the exact dose and how long its effects endure, when given locally.

In conclusion, gaseous administration of H_2_S protects the heart from IRI, likely through reduction of myocardial ROS production and the inhibition of inflammation, necrosis and fibrogenesis. Hypometabolism-inducing concentrations of H_2_S seem to have additional protective effects on necrotic cell death shortly after ischemia. H_2_S treatment might be of clinical use in myocardial ischemia or cardiac transplantation, where it could lead to reduced myocardial damage related to hypoxia.
